# Genotoxic effect of 2,2’-bis(bicyclo[2.2.1] heptane) on bacterial cells

**DOI:** 10.1371/journal.pone.0228525

**Published:** 2020-08-21

**Authors:** A. Kessenikh, E. Gnuchikh, S. Bazhenov, M. Bermeshev, V. Pevgov, V. Samoilov, S. Shorunov, A. Maksimov, L. Yaguzhinsky, I. Manukhov

**Affiliations:** 1 Moscow Institute of Physics and Technology, Dolgoprudny, Moscow, Russia; 2 State Research Institute of Genetics and Selection of Industrial Microorganisms of the National Research Centre “Kurchatov Institute”, Kurchatov Genomic Center, Moscow, Russia; 3 NRC “Kurchatov Institute”, Moscow, Russia; 4 Topchiev Institute of Petrochemical Synthesis, Russian Academy of Sciences, Moscow, Russia; 5 AN Belozersky Res Inst Physicochem Biol, Moscow MV Lomonosov State Univ, Moscow, Russia; VIT University, INDIA

## Abstract

The toxic effect of strained hydrocarbon 2,2'—bis (bicyclo[2.2.1]heptane) (BBH) was studied using whole-cell bacterial lux-biosensors based on *Escherichia coli* cells in which luciferase genes are transcriptionally fused with stress-inducible promoters. It was shown that BBH has the genotoxic effect causing bacterial SOS response however no alkylating effect has been revealed. In addition to DNA damage, there is an oxidative effect causing the response of OxyR/S and SoxR/S regulons. The most sensitive to BBH lux-biosensor was *E*. *coli* pSoxS-lux which reacts to the appearance of superoxide anion radicals in the cell. It is assumed that the oxidation of BBH leads to the generation of reactive oxygen species, which provide the main contribution to the genotoxicity of this substance.

## Introduction

**Fig 1:**
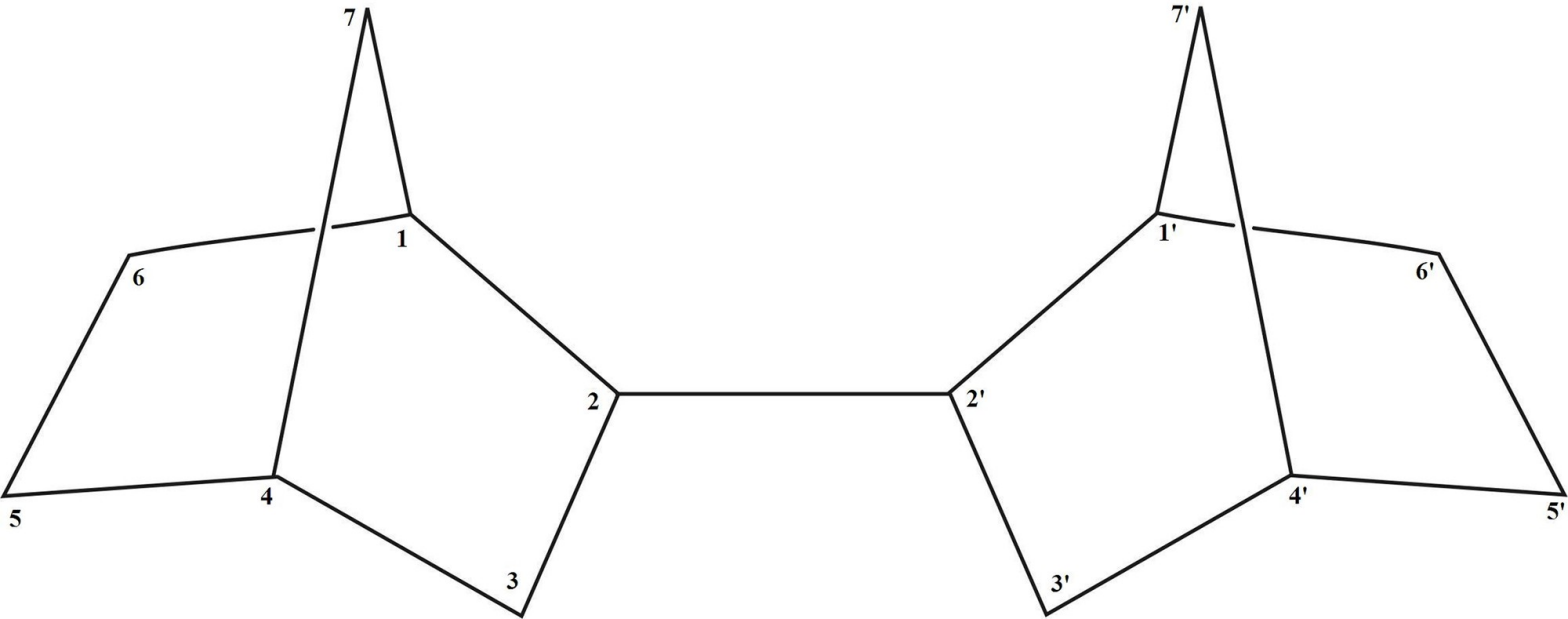
Structure of 2,2'-bis(bicyclo[2.2.1]heptane) molecule [4,5].

Strained hydrocarbons like norbornane and its non-saturated derivatives are commonly used in the production of rubber, epoxides, medicinal compounds and perfumes [1,2]. Notably, thermotechnical characteristics of strained hydrocarbons, which have extra internal energy due to deformation of valence bond angles, made them attractive for high-performance combustion applications [3]. Strained 2,2'-bis(bicyclo[2.2.1]heptane) (BBH) compound is a promising as a fuel component for liquid rocket engines. It is assumed that BBH is comparable with the unsymmetrical dimethylhydrazine (UDMH) in terms of specific impulse efficiency, but can be significantly less toxic to the environment and personnel working with rocketry. For the purpose of this study, BBH which consists of two strained structures made of 14 carbons and 22 hydrogens (Fig 1) was synthesized from 5-vinyl-2-norbornene [4–6]. Here we present the data describing the toxicity of BBH, which we had obtained by utilizing bacterial *lux*-biosensors. *Lux*-biosensors are living *Escherichia coli* cells transformed with hybrid plasmids, containing *luxCDABE* genes of *Photorhabdus luminescens* under control of various stress promoters, responsible for increasing the cells luminescence in occurrence of toxicants in the environment [7–11]. Similarly designed study of UDMH toxicity has been completed earlier [12,13].

The purpose of this work was to test genotoxicity of BBH and to investigate the mechanisms of genotoxicity if any occurs. The mechanism of BBH genotoxicity was evaluated using specific lux-biosensors of *E*. *coli* MG1655 cells with hybrid plasmids pAlkA-lux, pOxyR-lux, pSoxS-lux, and pColD-lux, reacting to DNA alkylation, oxidative damage by hydrogen peroxide, superoxide anion radicals and DNA damages that cause an SOS response, respectively. Threshold concentrations of BBH and UDMH were compared for different stress-responsive promoters.

## Materials and methods

### Chemicals

All chemicals were of analytical purity. Hydrogen peroxide was obtained from the firm "Ferraine" (Russia). Mitomycin C, N,N'-dimethyl-4,4'-dipyridyl dichloride (paraquat), methyl methanesulfonate, and enzyme catalase obtained from Sigma-Aldrich Co (USA). All test solutions were prepared immediately before use. The investigated compound 2,2 '—bis (bicycle [2.2.1] heptane) was synthesized by the Diels-Alder reaction from 5-vinyl-2-norbornene and dicyclopentadiene according to [14] with the subsequent stage of exhaustive hydrogenation of the cycloadduct in methanol on a Pd/C catalyst (1%) with hydrogen (25°C, 20 ATM, 24 h) [1,6].

### Bacterial strains and plasmids

The cells of *E*. *coli* K12 strain MG1655 F^-^ *ilv*G *rfb*-50 *rph*-1 (obtained from VKPM collection) were combined with plasmids pAlkA-lux, pColD-lux, pOxyR-lux and pSoxS-lux [10,11,15–17] that were built using a promoterless plasmid backbone pDEW201 [8]. pAlkA-lux carries P*alkA*, sensitive to DNA alkylation in cell. pColD-lux carries P*colD*, sensitive to DNA damages causing SOS-response. pOxyR-lux carries P*oxyR*, inducible by hydrogen peroxide or alkyl hydroperoxide. pSoxS-lux carries P*soxS*, inducible by superoxide anion radical. *E*. *coli* MG1655 cells transformed with pXen7 plasmid constitutively expressing *luxCDABE* genes [18] were employed as non-inducible control. Photos of luminescent colonies of *E*. *coli* MG1655 pXen7 are given in supplementary (S1 Fig in S1 File).

### Media and culturing conditions

Cell cultures were prepared by overnight cultivation at 37°C with aeration at 200 rpm in the 25 ml glass culture tubes in 5 ml of LB media supplemented with 100 μg/ml ampicillin, then diluted 1:100 in LB and grown at 37°C till reaching *OD*_*600*_ = 0.1–0.2, which corresponds to early logarithmic phase. The resulting cultures were used for further experiments and luminescence measurements.

### Experiment conditions and luminescence measurement

Bacterial cultures were divided into the 200-μl portions in separate wells of 96-well plate, and then 20 μl of tested compound (control toxicant or BBH in concentrations of 100, 10, or 1 mg/ml) were added. Then cells were incubated without shaking at room temperature with repetitive direct measurements of total bioluminescence (in RLU, relative light units) using plate luminometer LM–01A (Immunotech, Czech Republic). The measurement time depended on the type of biosensor used. The average common measurement time is 2 hours [10,12,13]. Since BBH is a new compound and has an unknown mechanism of interaction with cellular structures, preliminary experiments were extended by 2–3 hours to enable postponed effects detection. After characteristic response time was evaluated series of experiments were conducted with measurement times allowing to see the most significant biosensors signals. This time was 3 hours for pAlkA-lux, 2 hours for pOxyR-lux, 5 hours for pColD-lux, and pSoxS-lux. For threshold concentrations identification experiments with each biosensor were carried out with serial twofold BBH dilutions from 1 to 500 mM.

### Statistical analysis

Average values and standard deviations were calculated for five independent replications in each experiment. One-way ANOVA followed by Dunnett's multiple comparisons test and one-sample t-test for each concentration of test substance for all biosensors were performed using GraphPad Prism version 8.01 for Windows, GraphPad Software, La Jolla California USA, (www.graphpad.com). Area under curve (AUC) obtained by integrating kinetic curves data.

## Results

### Induction of P*alkA*

**Fig 2:**
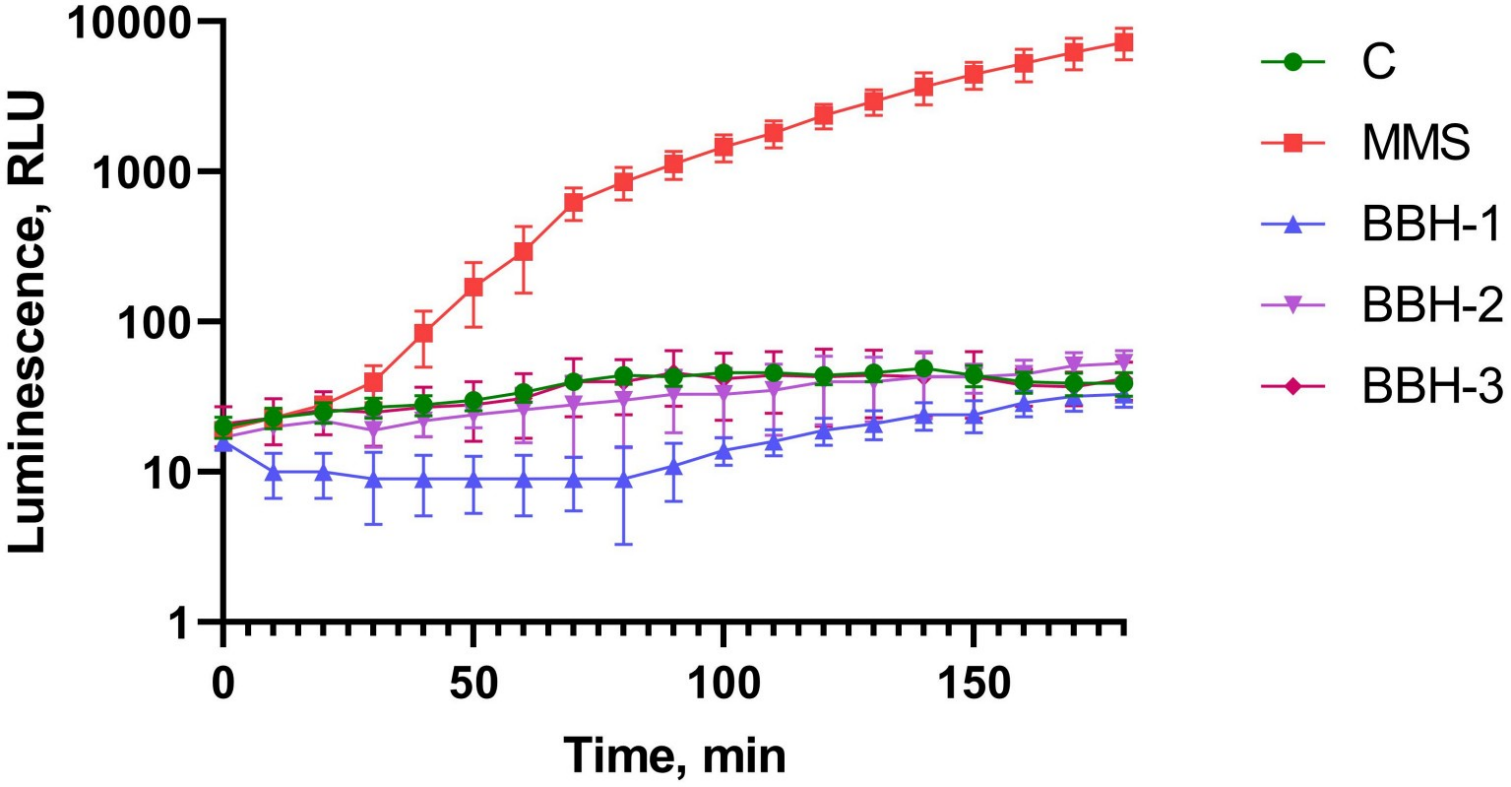
Luminescence of *E*. *coli* MG1655 pAlkA-lux cells after BBH addition depending on incubation time. c-control cells without toxicant addition, mms—MMS is added to final concentration of 100 μM. BBH-1- BBH is added to final concentration of 100 g/l, BBH-2–10 g/l, BBH-3–1 g/l.

At the first stage, the ability of BBH to alkylate DNA was investigated with use of *E*. *coli* MG1655 (pAlkA-lux) biosensor (Fig 2). The pAlkA-lux plasmid contains *lux*-genes under the control P*alkA*, therefore when alkylating agents occur in the sample the luminescence increases. Methyl methanesulfonate (MMS) in concentration of 100 μM was used as the alkylating substance in a positive control.

As one can see in Fig 2, none of the tested concentrations of BBH caused an alkylating effect as luminescence does not increase. One-way ANOVA for 1 and 10 g / l BBH and control curves gives a p-value of 0.35. At a maximum concentration of 100 g/l (10%) BBH has a cytotoxic effect, leading to a slight but still statistically significant decrease (p-value < 0.001, Dunnett’s test) in the background luminescence of cells (about 2–3 times).

### Induction of P*oxyR*

**Fig 3:**
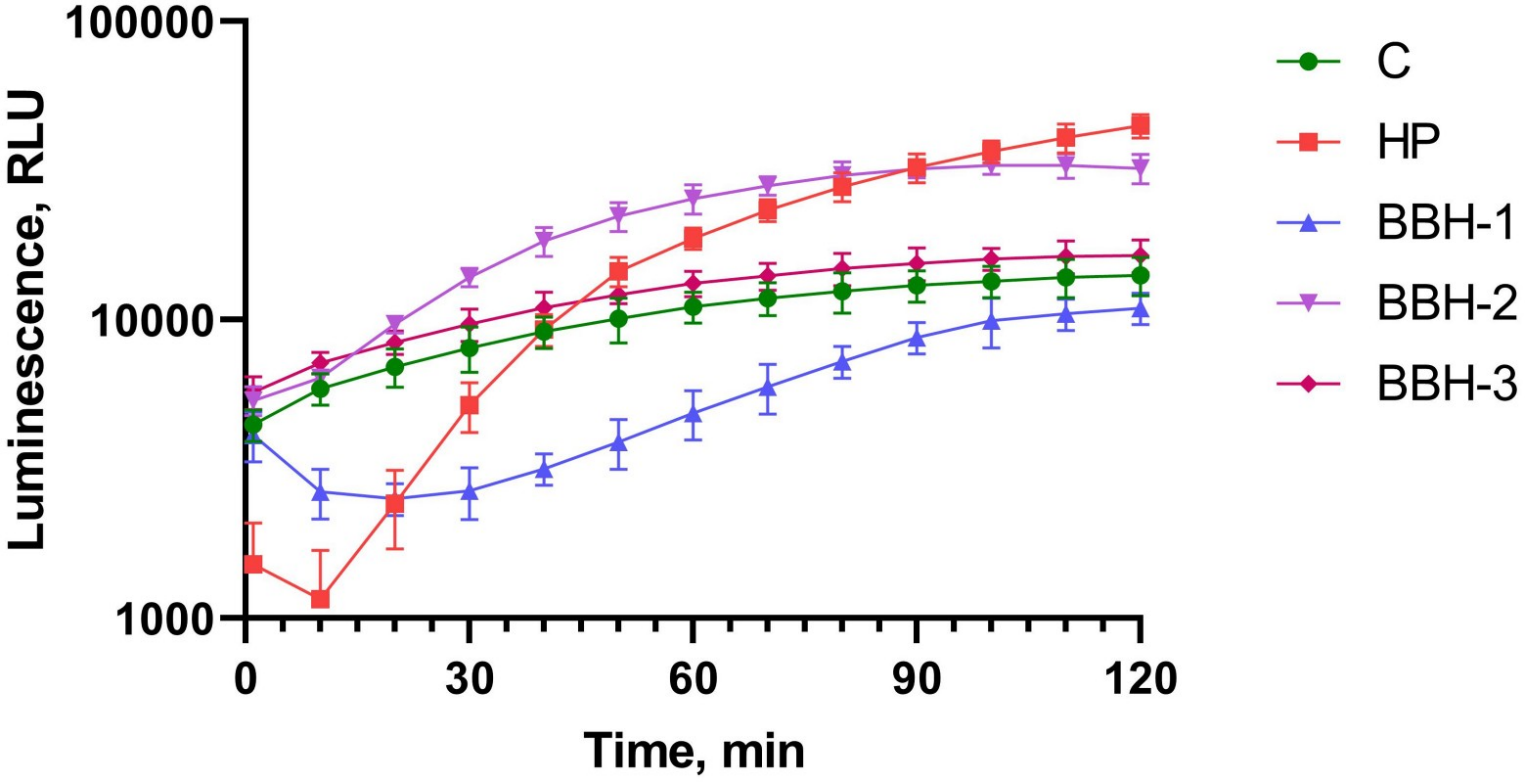
Luminescence of *E*. *coli* MG1655 (pOxyR-lux) cells after BBH addition depending on incubation time. c—control cells of *E*. *coli* MG1655 (pOxyR-lux) without toxicant addition, HP—hydrogen peroxide is added to a final concentration of 1 mM. BBH-1- BBH is added to final concentration of 100 g/l added, BBH -2-10 g/l, BBH -3–1 g/l, cat-2—BBH to final concentration of 10 g/l and catalase added.

Fig 3 shows the measurement of the luminescence kinetics of *E*. *coli* MG1655 (pOxyR-lux) after the addition of BBH. Hydrogen peroxide in concentration of 1 mM was used in a positive control.

BBH causes induction of the *oxyR* gene promoter (Fig 3). P*oxyR* activates in response to appearance of hydrogen peroxide or alkyl hydroperoxides. These toxicants can cause modifications of DNA nitrogenous bases, leading to an increase in the rate of mutagenesis [19,20]. The maximum effect is achieved at BBH 1% (Fig 3, curve BBH-2), a lower concentration does not cause a significant increase in luminescence, and a higher has a general toxic effect on cells, leading to a decrease in the base level of luminescence. One-way ANOVA for BBH (all concentrations) and control curves gives p-value < 0.001. In the presence of catalase, the induction of the P*oxyR* by 1% BBH was almost abolished.

### Induction of P*colD*

**Fig 4:**
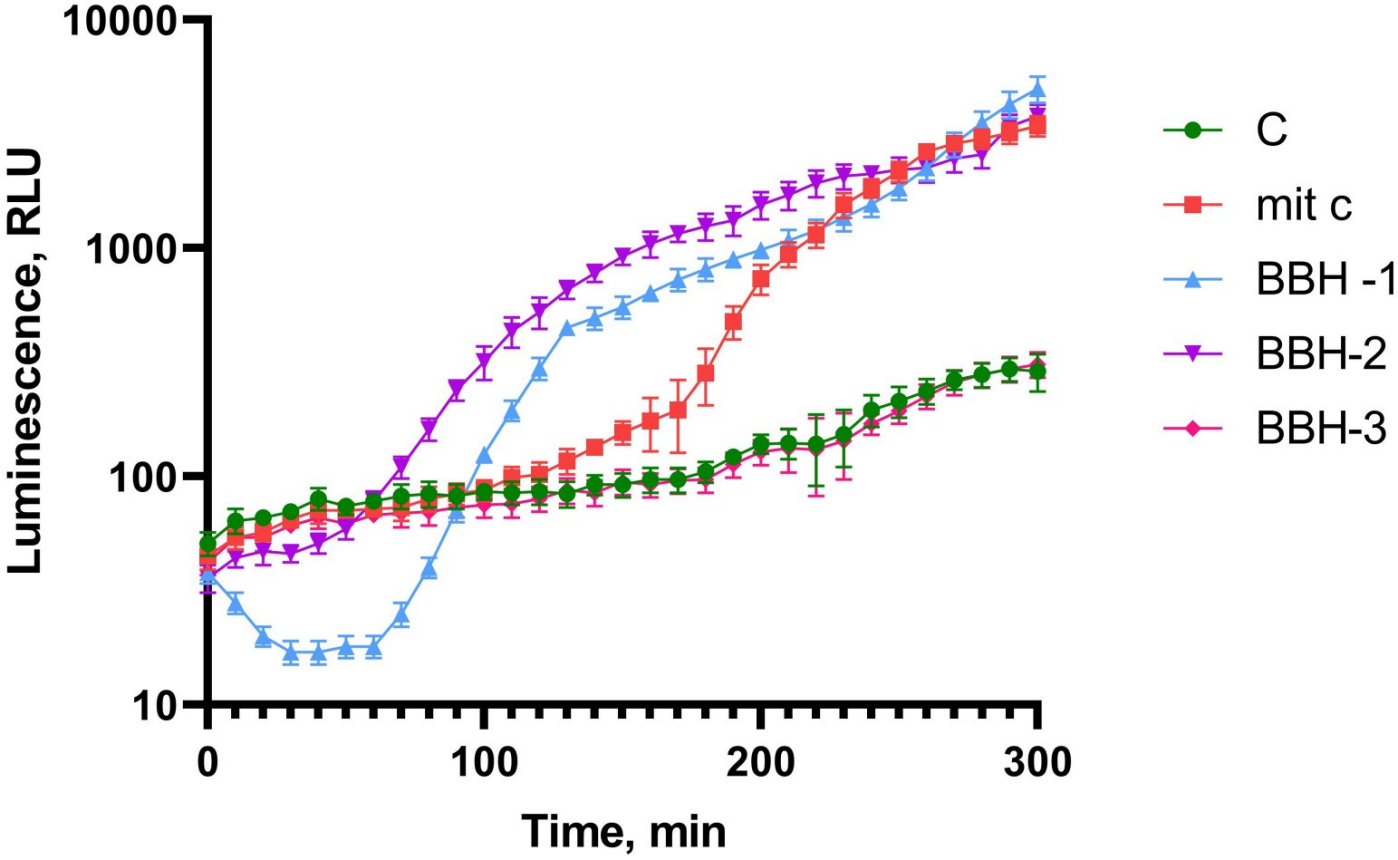
Luminescence of *E*. *coli* MG1655 pColD-lux cells after BBH addition depending on incubation time. c-*E*. *coli* MG1655 pColD-lux control cells without toxicant addition, mit c—mitomycin C is added to the final concentration of 10 μM. BBH-1—BBH added is to final concentration of 100 g/l, BBH-2–10 g/l, BBH-3–1 g/l.

Fig 4 shows the measurement of luminescence kinetics of *E*. *coli* MG1655 (pColD-lux) after the addition of BBH. The antibiotic mitomycin C was used in a positive control. It forms crosslinking with DNA, causes a stop of the replication fork, the formation of single-stranded DNA sites, and, as a consequence, SOS response.

As can be seen from the data shown in the Fig 4, the addition of BBH in concentrations of 10% and 1% leads to the high level of DNA damage, which causes an SOS response. The maximum possible response amplitude of the *E*. *coli* MG1655 (pColD-lux) biosensor is about three orders of magnitude [10]. In this experiment, we see the maximum biosensor activation of about 20 times during incubation with BBH at a concentration of 10 g/l. One-way ANOVA for BBH (all concentrations) and control curves gives p-value <0.001.

### Induction of P*soxS*

**Fig 5:**
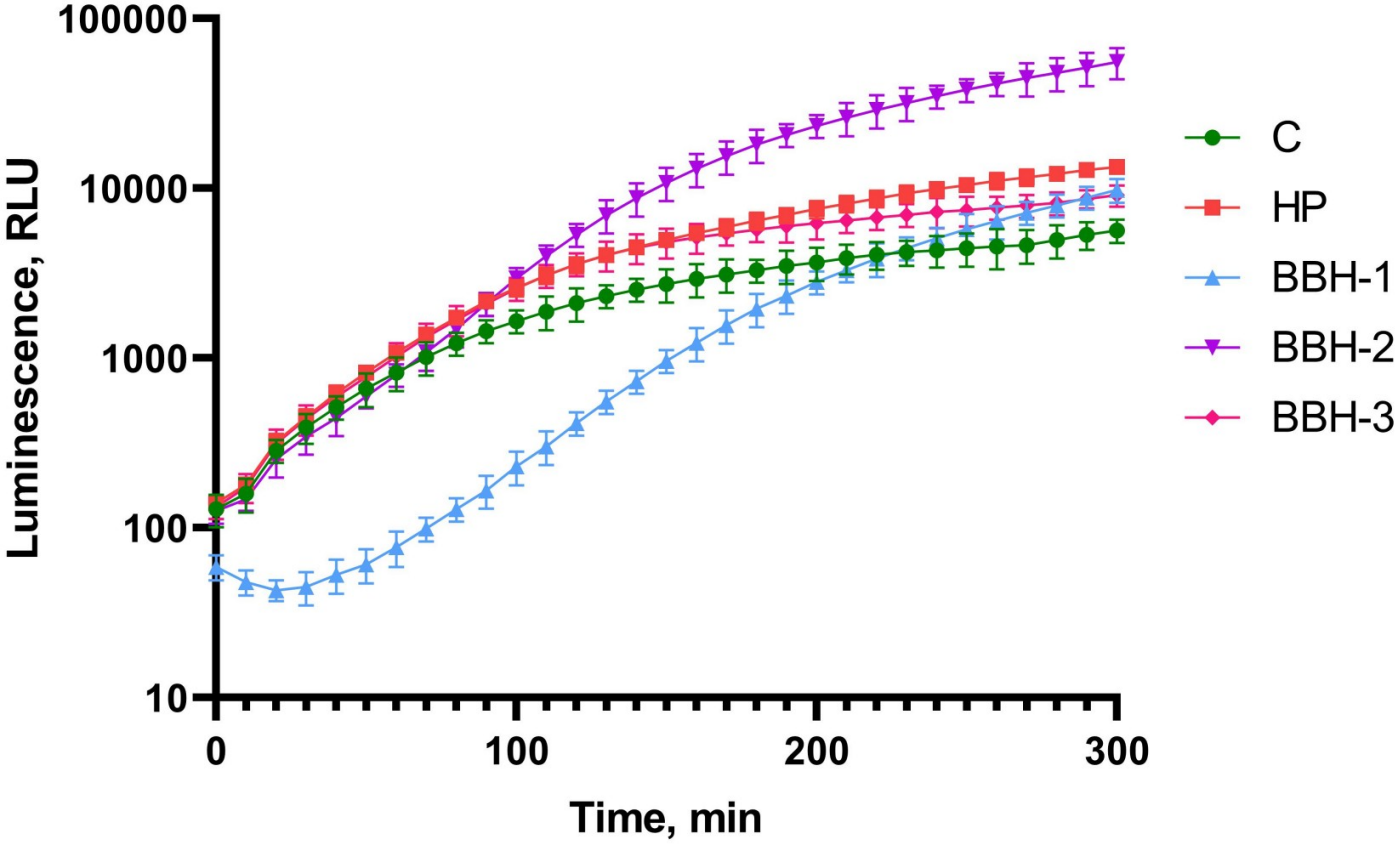
Luminescence of *E*. *coli* MG1655 pSoxS-lux cells after BBH addition depending on incubation time. c—control cells of *E*. *coli* MG1655 pSoxS-lux without toxicant addition, HP—hydrogen peroxide is added to a final concentration of 1 mM. BBH-1 –BBH is added to final concentration of 100 g/l, BBH-2–10 g/l, BBH-3–1 g/l.

Then the appearance of a superoxide anion radical in cells during incubation in the presence of BBH was investigated. For this purpose, *lux*-genes under the control of the P*soxS* promoter were used. Fig 5 shows the luminescence kinetics of *E*. *coli* MG1655 (pSoxS-lux) cells incubated with BBH. As a standard inductor for P*soxS* promoter paraquat is usually used–a substance that leads to occurrence of superoxide anion radical as a result of reactions with quinones of the respiratory chain. Hydrogen peroxide does not directly react with the SoxR protein but causes lipid peroxidation and the respiratory chain proteins damage that eventually leads to an increase in the superoxide anion radical pool in the cell [10,21]. Thus, H_2_O_2_ can also be used in a positive control to *E*. *coli* MG1655 (pSoxS-lux).

As one can see from Fig 5, activation of the P*soxS* promoter occurs when BBH is added at all concentrations from 1 to 100 g/l. One-way ANOVA for BBH (all concentrations) and control curves gives p-value < 0.001. On the basis of the data obtained in the experiments we can suggest that BBH causes the appearance of a superoxide anion radical in the cell.

### Comparative analysis of biosensors activation

**Fig 6:**
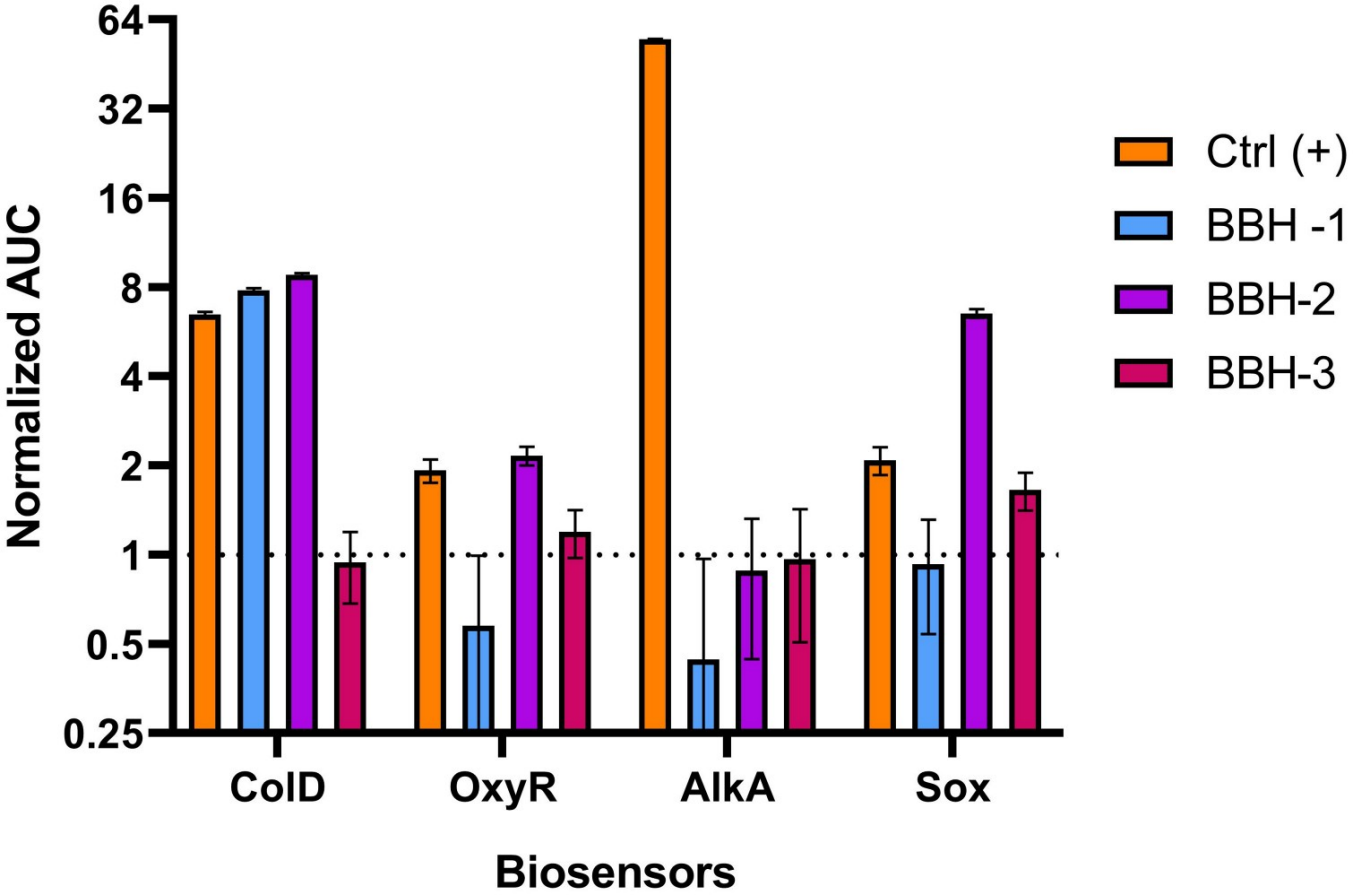
Dependence of biosensors activation on BBH concentration. The ordinate is the ratio of the area under curve for the test substance to the area under the control curve. Values marked * are significantly different from the control curve with P-value <0.05. Ctrl (+)—biosensor cells with the addition of a standard toxicant specific to each biosensor, BBH-1 –BBH is added to final concentration of 100 g/l, BBH-2–10 g/l, BBH-3–1 g/l.

The diagram in Fig 6 demonstrates the activation of biosensors with different BBH concentrations. Activation values were determined as ratio of the area under the curve for each concentration of the test substance to the area under the control curve without the addition of toxicant. Areas (AUC) are obtained by integrating the kinetic curve data shown in graphs 2–5. Statistical analysis was performed by Student's one-sample test.

As can be seen from the diagram in Fig 6, the BBH concentration 100 g/l, having a significant general toxicity for cells, reduces the luminescence of all used biosensors. Only *E*. *coli* MG1655 pColD-lux activated by this concentration of BBH exceeds the control curve (after 1.5 hours). This occurs, despite partial cell death, in virtue of the high induction amplitude of the biosensor with the P*colD* promoter (up to 20 times). A BBH concentration 10 g/l causes a statistically significant activation of the P*colD*, P*soxS*, and P*oxyR* promoters (p < 0.001, one sample t-test), but not the P*alkA* promoter. The BBH concentration 1 g/l causes a statistically significant activation only of the P*soxS* promoter (p = 0.02, one sample t-test).

### Threshold concentrations for lux biosensors

Table 1 shows the threshold concentrations of UDMH and BBH possible to activate stress promoters. As control, data are given for standard, promoter-specific toxicants inducing a noticeable (1.5–2 times) effect of bioluminescence enhancement of *lux*-biosensors. Mitomycin C induces damage of DNA, hydrogen peroxide causes the oxidative stress, paraquat induces oxidative stress through generation of superoxide anion radicals, methylnitronitrosoguanidine (N-methyl-N'-nitro-N-nitrosoguanidine) (MNNG) alkylates DNA.

**Table 1:** Threshold concentrations for lux biosensors.

Biosensor	UDMH* mM	BBH, mM	Standard toxicant, mM	Note
pAlkA-lux	2×10^−2^	nd**	MNNG, 10^−5^	Alkylation of DNA
pOxyR-lux	3×10^−3^	10±3.5	H_2_O_2_, 2x10^-4^	Oxidation by hydrogen peroxide [22]
pColD-lux	8×10^−3^	7±2.5	Mitomycin C, 10^−6^	DNA damages which are leads to the formation of single-stranded sections of DNA in the cell
pSoxS-lux	0.2	3±1.4	paraquat, 10^−4^	Oxidation by superoxide anion radicals
pXen7	2	40±12	C_2_H_5_OH, 200	Total toxicity is measured by the incidence of luminescence, which correlates with the number of living cells

* The values of threshold concentrations for UDMH and standard toxicants which were obtained by us earlier [23,24],

** nd—not determined.

## Discussion

It is known that degrading of a number of hydrocarbon compounds leads to reactive oxygen species generation and oxidative stress induction in bacterial cells [25,26].

According to the literature, BBH must be oxidized by oxygen, as well as all hydrocarbons, by a free-radical chain mechanism [27]. In [27] it was shown on the example of cyclopropane that hydroxyl radical is one of the products of strained compounds destruction under intense light. The BBH molecule includes two elements with strained bonds, which determine a higher energy release in the oxidation process compared to conventional non-strained hydrocarbons. In [3] comparison of ΔH^≠^ (in Kcal/mol) obtained for ring opening of cycloalkanes provided and the ring strain energy is shown to be 3.57 times higher in cyclobutanes compared to cyclopentane. The radical mechanism of oxidation along with the increased reaction energy suggests that the toxic effect of this type of compounds should be determined mainly by reactions of formation of reactive oxygen species. An increased content of superoxide anion radical in the cell can lead to DNA damage [28–30]. In this regard, it should be noted that according to our experiment, the toxicity of BBH, determined by the appearance of a superoxide anion radical in cells, is close to that of UDMH, despite the fact that the tests were conducted with an undispersed form of the product insoluble in water, whose contacts with biological objects are sharply reduced and are determined only by the interface of the phases. Under natural conditions, deep dispersion of products is completely absent during fuel spillage, including a humid environment. Thus, the chosen experimental conditions achieve convergence with the conditions of accidental fuel spill.

The genotoxic effect of BBH definitely takes place and is expressed by cell damages that activates the following defense systems: SOS response, *oxyR/S* regulon, and *soxR/S* regulon.

The time for the appearance of H_2_O_2_ in quantities sufficient to activate the cellular response systems after adding BBH to the medium is rather short and is expressed in a minute scale as the P*oxyR* response to BBH 10 g/l is comparable in speed with the positive control, where 1 mM H_2_O_2_ was added at the beginning of incubation. It takes more time for P*colD* to respond: 50 minutes—induction onset (T_min_), 3.5 hours—maximum response (T_max_). This is consistent with the data obtained earlier for other genotoxic substances and is explained by the time spent on entering into the cell, DNA damaging that blocks replication [31], formation of single strand ends and activation of SOS response [10,32]. The long time required for the P*soxS* response (T_min_ = 70 min, T_max_ = 4 hours) is apparently explained by the fact that the appearance of superoxide anion radical in the cell is obviously associated only with damage of the respiratory chain [33]. Respiratory chain damage is possible as a result of exposure to alkyl radicals or H_2_O_2_ arising from the oxidation of BBH. However, the concentration of H_2_O_2_ is significantly less than 1 mM and cannot affect the P*soxS* promoter. The permeability of the cell membrane for alkyl radicals arising from BBH oxidation was not investigated before and apparently takes time.

Activation of SOS response occurs only at very high concentrations of BBH in the medium (threshold concentration is about 1 g/l). The test using *E*. *coli* MG1655 (pColD-lux) is more sensitive to genotoxic agents than SOS chromotest [34], which is used in toxicology along with the Ames test [35,36] to determine the rate of mutagenesis. These tests correlate well with each other [32], but may underestimate the effect of alkylating compounds on the rate of mutagenesis [37]. In the present work, using the *E*. *coli* MG1655 (pAlkA-lux) biosensor, it was shown that during incubation of cells with BBH, DNA alkylation is not observed and, obviously, alkylating compounds do not appear in the medium.

The oxidation of BBH under aerobic conditions in the presence of *E*. *coli* cells (Figs 3 and 5, Table 1) leads to the appearance of superoxide anion radical and hydrogen peroxide. As a result, alkyl radicals or alkylhydroperoxides can occur in an organic-rich environment. The addition of catalase to the medium significantly reduces the activation of the *oxyR* promoter (Fig 3, curve cat-2), which indicates the appearance of hydrogen peroxide, which easily penetrates the cell membrane. Hydrogen peroxide and superoxide radicals, as well as possible alkyl radicals and alkyl hydroperoxides, cause the oxidation of the nitrogenous bases of DNA which accelerates mutagenesis as a result of the inaccurate activity of the DNA polymerase [19,20,28–30].

## Conclusion

We believe that the basis of the genotoxic effect of BBH on cells are provided by reactive oxygen species that damage the DNA therefore accelerating mutagenesis. Thus, the mechanism of genotoxic action of BBH is fundamentally different from the action of UDMH, which is determined by alkylating derivatives, primarily nitrosodimethylamine [13] and superoxide-anion radicals arising from the oxidation of UDMH with atmospheric oxygen [12].

## Supporting information

S1 File (DOC)

## Abbreviations

BBH: 2,2'-bis(bicyclo[2.2.1]heptane)
MNNG: N-methyl-N'-nitro-N-nitrosoguanidine
RLU: relative light units
UDMH: unsymmetrical dimethylhydrazine
