# Correction: Genotoxic effect of 2,2’-bis(bicyclo[2.2.1] heptane) on bacterial cells

**DOI:** 10.1371/journal.pone.0248251

**Published:** 2021-03-03

**Authors:** A. Kessenikh, E. Gnuchikh, S. Bazhenov, M. Bermeshev, V. Pevgov, V. Samoilov, S. Shorunov, A. Maksimov, L. Yaguzhinsky, I. Manukhov

There are errors in the author affiliations. The correct affiliations are as follows:

A. Kessenikh^1^, E. Gnuchikh^1,2,3^, S. Bazhenov^1,6^, M. Bermeshev^4^, V. Pevgov^1^, V. Samoilov^4^, S. Shorunov^4^, A. Maksimov^4^, L. Yaguzhinsky^1,5^, I. Manukhov^1,6,7^

1 Moscow Institute of Physics and Technology, Dolgoprudny, Moscow, Russia, 2 State Research Institute of Genetics and Selection of Industrial Microorganisms of the National Research Centre “Kurchatov Institute”, Kurchatov Genomic Center, Moscow, Russia, 3 NRC “Kurchatov Institute”, Moscow, Russia, 4 Topchiev Institute of Petrochemical Synthesis, Russian Academy of Sciences, Moscow, Russia, 5 AN Belozersky Res Inst Physicochem Biol, Moscow MV Lomonosov State Univ, Moscow, Russia, 6 HSE University, Moscow, Russia, 7 Federal Research Center of Biological Systems and Agro-technologies of RAS, Orenburg, Russia.
