# Supplementary material for: Genotoxic effect of 2,2’-bis(bicyclo[2.2.1] heptane) on bacterial cells
Source: PLoS One. 2020 Aug 21;15(8):e0228525. doi: 10.1371/journal.pone.0228525 (PMC7444485; doi:10.1371/journal.pone.0228525)
Supplement: S1 File — (DOC) [file pone.0228525.s001.doc]

Genotoxic effect of 2,2’-bis(bicyclo[2.2.1] heptane) on bacterial cells.

Kessenikh A. G.^1^, Gnuchikh E. Yu.^1,2,3^, Bazhenov S. V.^1^, Bermeshev M. V.^4^, Pevgov V. G.^1^, Samoilov V. O.^4^, Shorunov S. V.^4^, Maksimov A. L.^4^, Yaguzhinsky, L. S.^1,5^, Manukhov I. V.^1,2,*^

^1^Moscow Institute of Physics and Technology. Dolgoprudny, Moscow region, Russia.

^2^State Research Institute of Genetics and Selection of Industrial Microorganisms of the National Research Centre «Kurchatov Institute», Kurchatov Genomic Center, Moscow 117545, 1st-Dorozhnii pr. 1, Russia.

^3^NRC «Kurchatov Institute», Moscow, 123182, Akademika Kurchatova pl., 1, Russia.

^4^Topchiev Institute of Petrochemical Synthesis. Russian Academy of Sciences, Moscow, Russia.

^5^Moscow MV Lomonosov State Univ, AN Belozersky Res Inst Physicochem Biol, Moscow, Russia.

**Corresponding author contacts**

Ilya V. Manukhov, [manukhovi@mail.ru](mailto:manukhovi@mail.ru), +7 905 562 2924

Biosensor cells *E. coli* MG1655 pXen7, used to determine the general toxicity of BBH, contain *lux* genes under the control of a constitutive promoter and luminesce 3-4 orders of magnitude stronger than inducible biosensors. Unfortunately, the basic intensity of bioluminescence of lux-biosensor cells with promoters P*colD,*  P*soxS,* P*oxyR* and P*alkA* is not sufficient for photographing. We tried to get images from a microscope (Widefield epifluorescence images were acquired with an inverted microscope (Elyra, Zeiss) using Apochromat 100x oil immersion objective (NA=1.46, Zeiss) and iXon 897 EMCCD camera (512x512 px, Andor)) for which the manufacturer claims this possibility. However, the declared sensitivity is not enough for photographing luminescent lux-biosensors. Although the signal is sufficient to detect it using a scintillation counter or using a photomultiplier in the luminometers specified in the materials and methods. We can only provide photos of bright luminescent colonies of bacteria cells carrying luciferase genes are under a strong promoter (such as P*lac*) where the intensity of luminescence and the density of cells are sufficient for photographing. In a liquid medium at OD=0.1 with not the strongest promoters, it is not possible for us to obtain micrographs of luminescent cells.

**(A)**


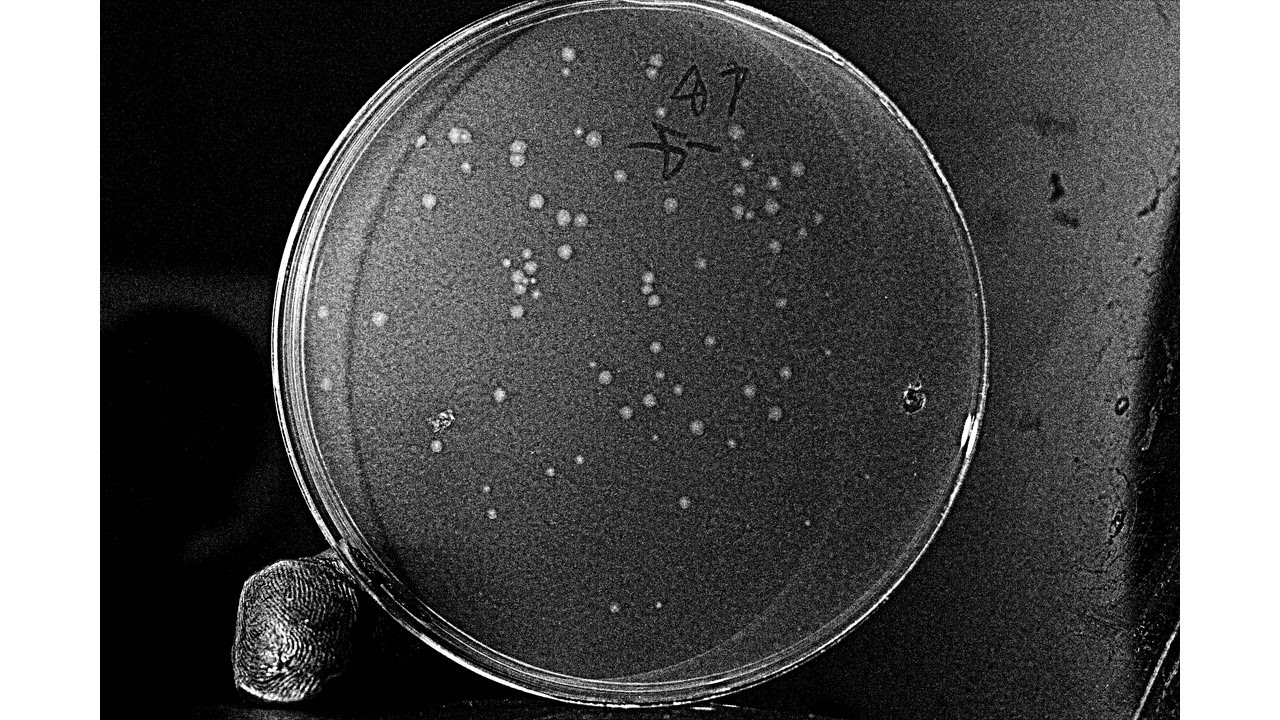


**(B)**


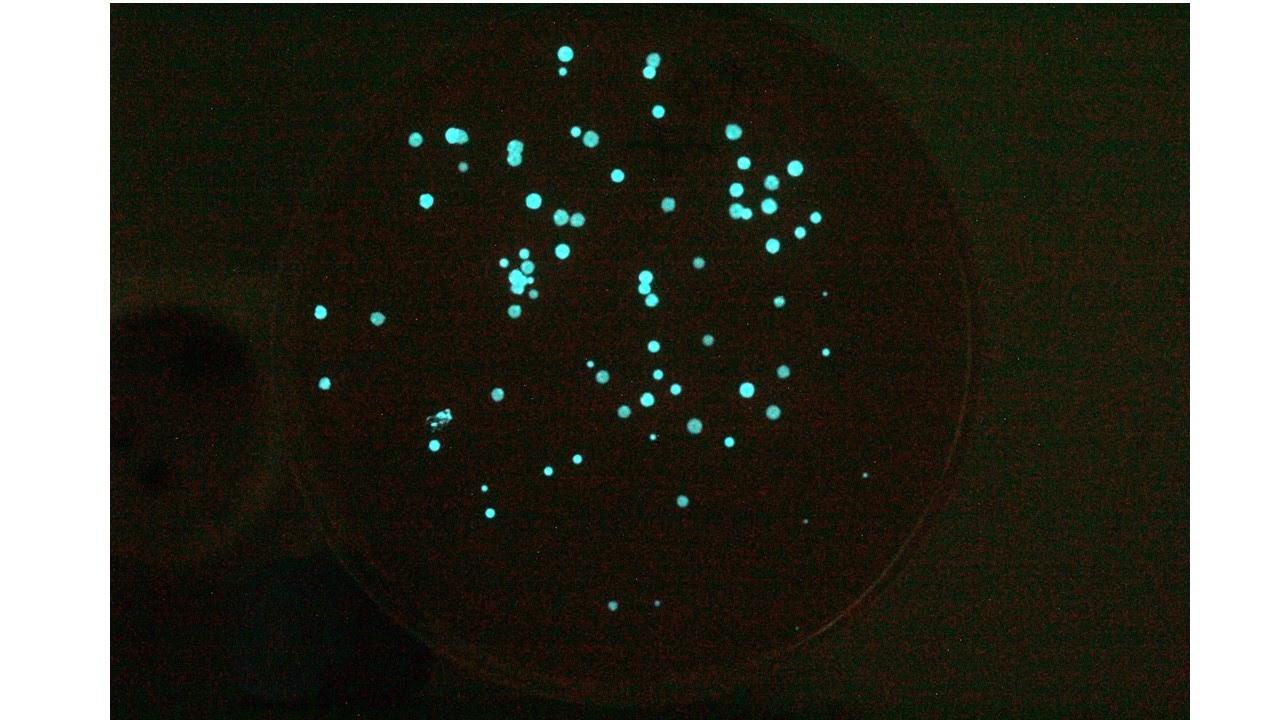


**Fig** **S1 Colonies of *E. coli* strain MG1655 pXen7 grown on agar medium are photographed with light (A) and without it (B).**
